# Colonic in vitro fermentation of mycoprotein promotes shifts in gut microbiota, with enrichment of *Bacteroides* species

**DOI:** 10.1038/s42003-024-05893-4

**Published:** 2024-03-05

**Authors:** Raffaele Colosimo, Hannah C. Harris, Jennifer Ahn-Jarvis, Perla Troncoso-Rey, Tim J. A. Finnigan, Pete J. Wilde, Frederick J. Warren

**Affiliations:** 1 Quadram Institute Bioscience, Norwich Research Park, Norwich, Norfolk NR4 7UQ UK; 2 Marlow Foods Ltd, Station Road, Stokesley, North Yorkshire TS9 7AB UK; 3 Present Address: Elsevier B.V, Radarweg 29a, 1043 NX Amsterdam, Netherlands

**Keywords:** Metagenomics, Microbiome

## Abstract

Mycoprotein is a fungal-derived ingredient used for meat alternative products whose fungal cell walls are rich in dietary fibre (β-glucans and chitin) and defines its structure. Several health benefits have been reported after mycoprotein consumption, however, little is known about the impact of mycoprotein fermentation on the gut microbiota. This study aims to identify changes in microbiome composition and microbial metabolites during colonic fermentation of mycoprotein following simulated upper gastrointestinal digestion. Changes in microbial populations and metabolites produced by the fermentation of mycoprotein fibre were investigated and compared to a plant (oat bran) and an animal (chicken) comparator. In this model fermentation system, mycoprotein and oat showed different but marked changes in the microbial population compared to chicken, which showed minimal differentiation. In particular, *Bacteroides* species known for degrading β-glucans were found in abundance following fermentation of mycoprotein fibre. Mycoprotein fermentation resulted in short-chain fatty acid production comparable with oat and chicken at 72 h. Significantly higher branched-chain amino acids were observed following chicken fermentation. This study suggests that the colonic fermentation of mycoprotein can promote changes in the colonic microbial profile. These results highlight the impact that the unique structure of mycoprotein can have on digestive processes and the gut microbiota.

## Abstract (web summary)

An in vitro model of upper gastrointestinal digestion approach identifies changes in microbiome composition and microbial metabolites during simulated colonic fermentation, and implicates several *Bacteroides* species in the process.

## Introduction

Mycoprotein (MYC) is the base ingredient in various alternative meat products that are obtained by the continuous fermentation of the filamentous fungus *Fusarium venenatum* A3/5 (ATCC PTA-2684). Production steps involve fermentation, heating to reduce RNA content, centrifugation, and freezing. A key component of MYC, which also defines its structure, is the fungal cell wall, mainly composed of dietary fibre (~2/3 β-glucans and 1/3 chitin)^1^. Previous studies have shown that the fungal cell wall of MYC appeared to be a crucial component in slowing down simulated upper GI digestion processes. The fibrous cell wall has been shown to entrap digestive components such as α-amylase^2^ and bile salts^3^. The entrapment of enzymes and sustained digestion of nutrients may reflect in vivo the homoeostasis and digestion of lipids and carbohydrates and explain the health effects related to cardiovascular diseases and type-2 diabetes observed after MYC consumption^4–8^.

Metabolites such as short-chain fatty acids (SCFAs), derived from dietary fibre fermentation in the lower gastrointestinal tract, and changes in the colonic microbial population can also modulate lipid and glucose metabolism. SCFAs have been reported to promote human health by regulating blood pressure, appetite, glucose homoeostasis and maintaining gut integrity^9^. Similarly, the gut microbiota composition has shown evidence of its link with metabolic disorders such as cardiovascular diseases and type-2 diabetes^10^, and specific bacterial species such as *Bacteroides* can help maintain gut microbial homoeostasis^11^.

A recent study has reported the production of SCFAs during colonic in vitro fermentation of the whole MYC and isolated MYC fibre^12^. However, complete simulated digestion of MYC, starting from the upper gastrointestinal tract (gastric and small intestinal step) followed by colonic fermentation, has never been investigated. Moreover, changes in the microbial population induced by MYC fermentation and specific bacterial species involved in the fermentation have not been investigated to date.

Thus, this paper investigates the colonic in vitro fermentation of MYC previously digested in a simulation of the upper gastrointestinal tract and compares it to an oat (OAT) and chicken (CKN) comparator. OAT and CKN can be fermented to produce SCFAs and modulate changes in the colonic bacterial population^13–15^. This comparison allows us to observe how substrates with structural differences (e.g., plant cell wall in OAT, fungal cell wall in MYC, and no cell wall in CKN) and belonging to distinct kingdoms of life (i.e., fungi, plants, and animals) could influence microbial changes and metabolite production. Indeed, SCFA production can vary depending on bacterial groups and substrate^16^; therefore, different dietary fibre substrates can promote distinct changes in the gut microbiota. Moreover, alterations in the MYC structure after a complete upper and lower simulated GI digestion were investigated.

## Results

### Differentiation of bacterial communities during colonic in vitro fermentation is driven by substrate

**Fig. 1:**
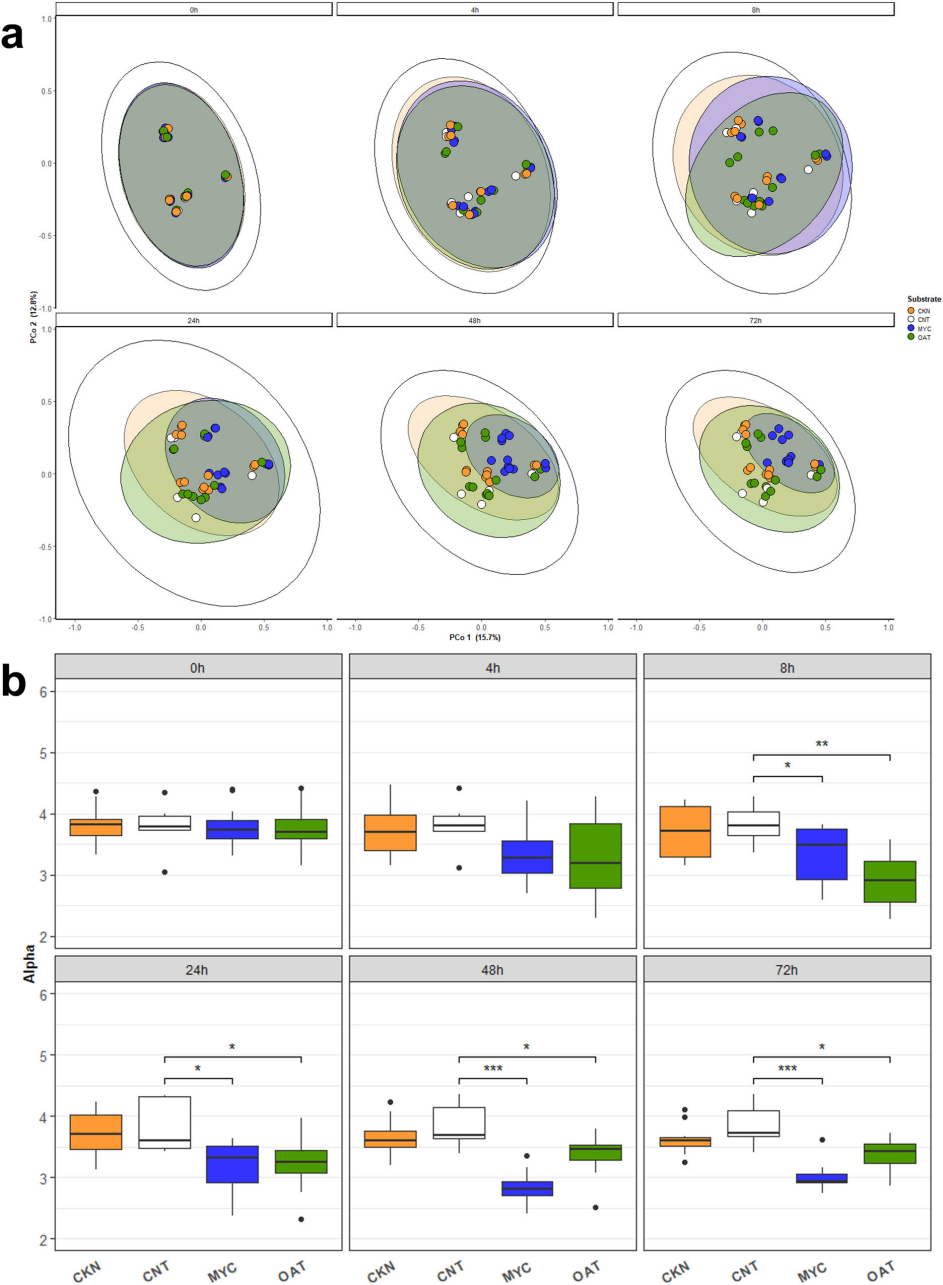
Microbial differentiation during colonic in vitro fermentation. **a** PCoA plot based on Bray–Curtis dissimilarity distance from the taxonomic composition at species-level estimated by MetaPhlAn, where the samples are grouped for timepoints (0, 4, 8, 24, 48, 72 h) of colonic in vitro fermentation of CNT, MYC, OAT, and CKN. **b** Changes in microbial community alpha diversity (Shannon index) for CNT, MYC, OAT and CKN grouped for timepoints (0, 4, 8, 24, 48, 72 h). Statistical significance testing was carried out with a Wilcoxon test relative to the CNT sample, and *p* values were corrected for multiple comparisons with the Benjamini-Hochberg method. *p ≤ 0.05, **p ≤ 0.01, ***p ≤ 0.001.

Microbiome community composition was analysed using MetaPhlAn4 (Supplementary Data 1 and 2). Figure 1a shows that the 0 h of all samples are clustered by donor and have similar starting microbiome composition irrespective of the substrate. At 72 h, the substrates differ from each other by showing a contrasting development compared to the 0 h. PERMANOVA analysis demonstrated that at 72 h there was a statistically significant (*p* = 0.001, F-statistic 4.89) difference in the microbial community between the substrates. Subsequent pairwise adonis analysis between substrates demonstrated that at 72 h the community composition for the MYC substrate was significantly different from the CNT (*q* = 0.0015, F-statistic 8.52), CKN (*q* = 0.0015, F-statistic 4.54) and OAT (*q* = 0.0015, F-statistic 5.26).

Figure 1b shows the changes in alpha diversity (Shannon index) during the course of the fermentation. At baseline, there are no significant differences between the substrates. From 8 h onwards there are significant differences between the CNT and the MYC and OAT substrates, reflecting the shifts in community composition observed in Fig. 1b. The CKN substrate was not significantly different to CNT at any timepoint. The CNT samples, which lacked a carbon source in the media, did not show significant changes in microbial community composition at any of the timepoints sampled.

### Species-level changes in microbiome composition are substrate-dependent

**Fig. 2:**
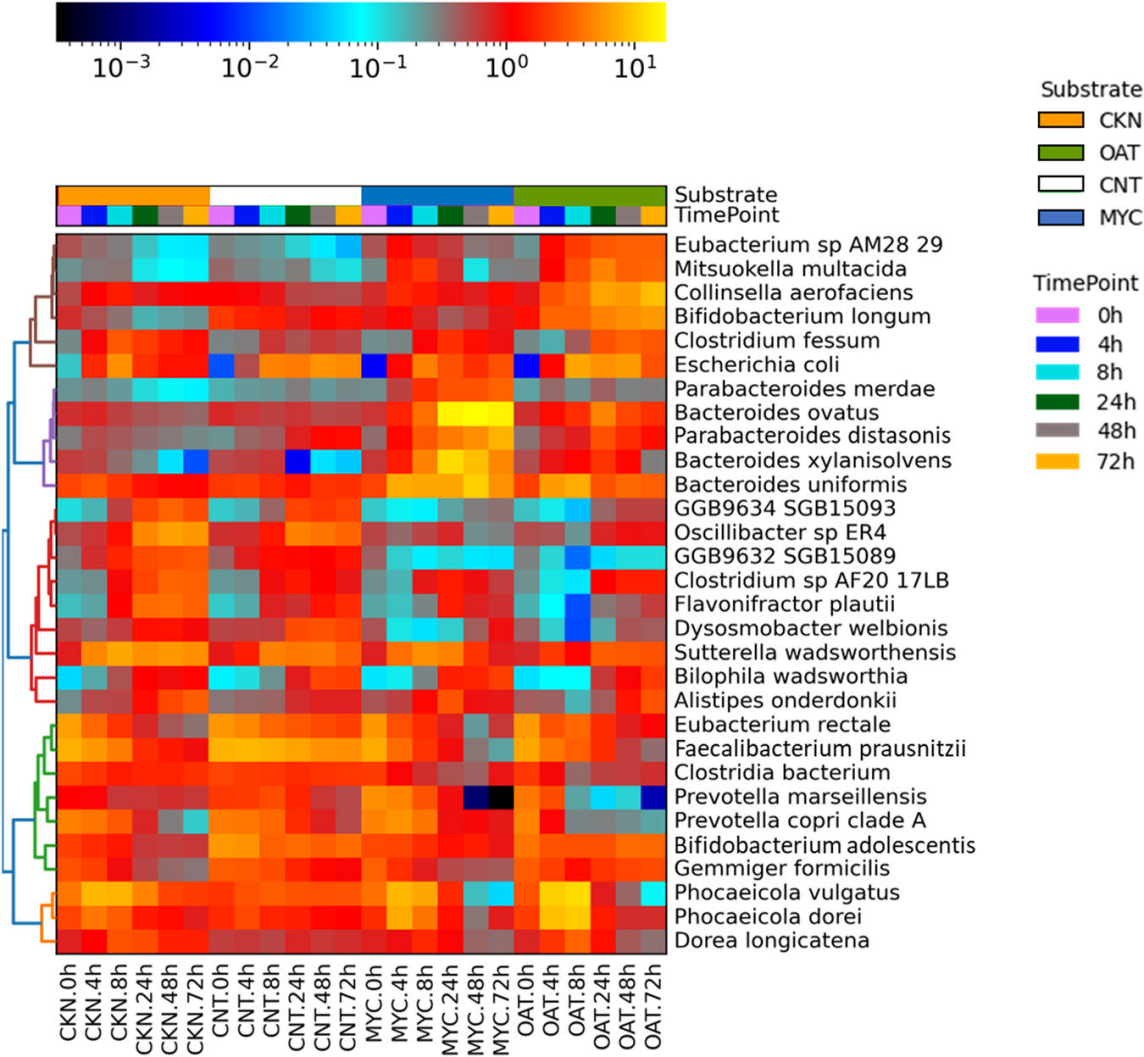
Heatmap showing the relative abundance for the 30 most abundant species. Abundances were shown for fermentation of CNT, MYC, OAT, and CKN during colonic in vitro fermentation (0, 4, 8, 24, 58, and 72 h).

Taxonomic analysis of the most abundant species during colonic in vitro fermentation of CNT, MYC, OAT, and CKN is shown in Fig. 2. This analysis revealed that the microbial population at 0 h had a high overall abundance of *Prevotella copri, Faecalibacterium prausnitzii and Eubacterium rectale* in MYC, OAT, CKN, and CNT. After 24 h of fermentation, distinct substrate-driven differences appear in the species-level microbial community composition. For the MYC samples, there are increases in abundance of several species of the genus *Bacteroides*, including *Bacteroides ovatus, Bacteroides uniformis* and *Bacteroides xylanisolvens*, as well as *Parabacteroides distasonis*. The increase in abundance in several species of the genus *Bacteroides* is reflected in the altered Bacteroidetes/Firmicutes ratio for the MYC fermentations. As shown in Supplementary Note 4, Figure S1, from 8 h onwards the Bacteroidetes/Firmicutes ratio for MYC is significantly higher than control, while the Bacteroidetes/Firmicutes ratio for the OAT and CKN remains unchanged. In contrast, the OAT substrate had lower levels of *Bacteroides* species, but higher levels of *Bifidobacterium*, specifically *Bifidobacterium longum* and *Bifidobacterium adolescentis*, as well as increased abundance of *Collinsella aerofaciens*. The CKN samples remained more similar to the 0 h timepoint throughout, with a small increase in *Alistipes onderdonkii* and *Sutterella wadsworthensis*.

### Substrate drives functional changes in microbiome metabolic pathway abundance

**Fig. 3:**
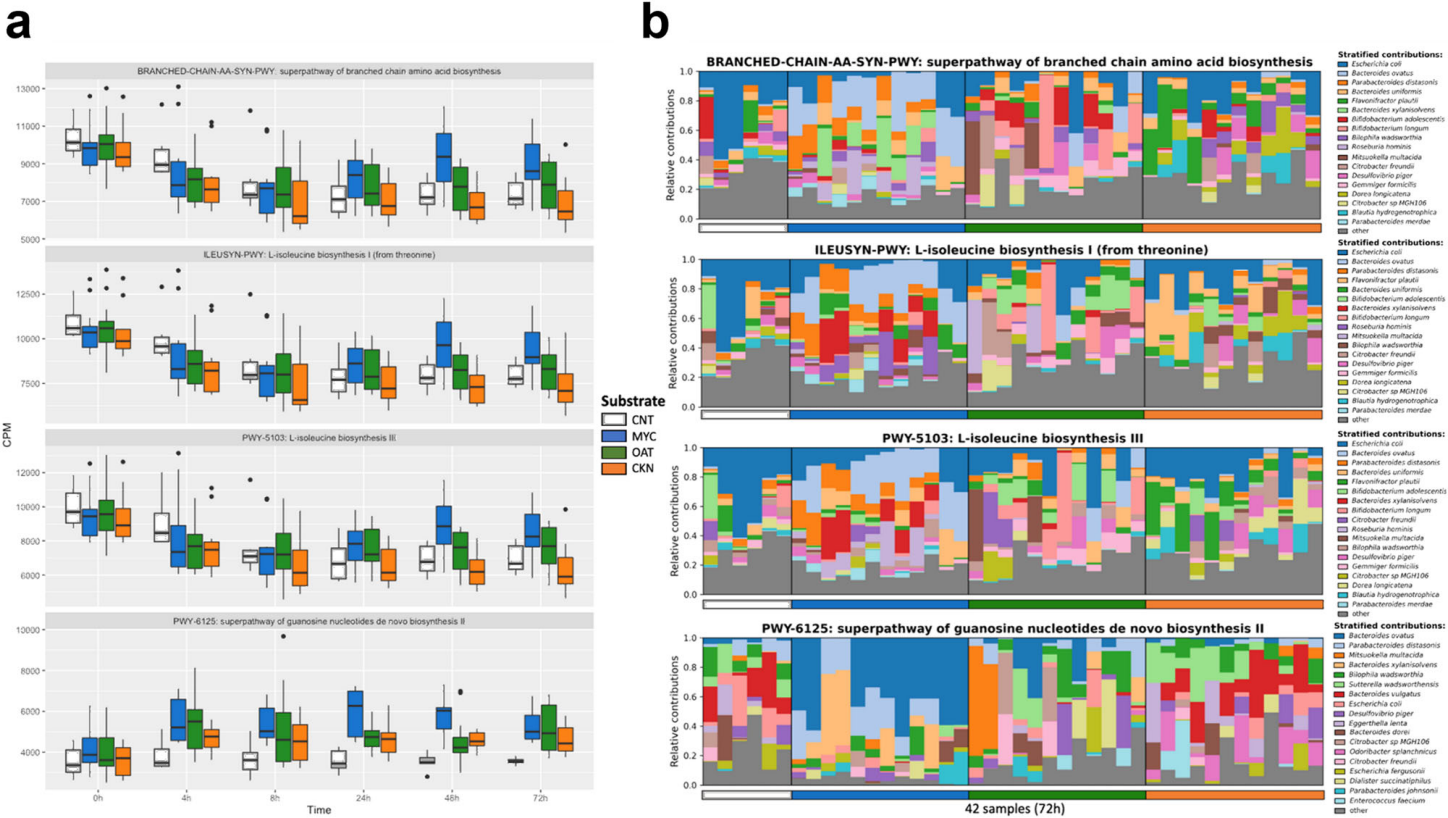
Metabolic pathways with the most significant increases in MYC. **a** The abundance in CPM for the pathways which were most enriched in MYC, relative to the other substrates. **b** the stratified taxa contribution for the pathways Superpathway of branched-chain amino acid biosynthesis; L-isoleucine biosynthesis I; L-isoleucine biosynthesis III; and Superpathway of guanosine nucleotides de novo biosynthesis II.

Supplementary Data 3 and 4 show multivariable association with linear models (MaAsLin2) analysis of the most statistically significant associations of specific pathways during 72 h of colonic fermentation with the three different substrates and the control. Figure 3 highlights the pathways which were most significantly associated with MYC fermentation. The Superpathway of branched-chain amino acid biosynthesis; L-isoleucine biosynthesis I; L-isoleucine biosynthesis III; and Superpathway of guanosine nucleotides de novo biosynthesis II were the most prevalent pathways correlated with MYC colonic fermentation. These metabolic pathways are central metabolic pathways present in *Bacteroides* species and reflect the high abundance of *B. ovatus, B.xylanisolvens,* and *B. uniformis* during MYC fermentation. Their association with MYC fermentation reflects the increases in abundance of these species in response to MYC fermentation. The CNT samples, lacking a carbon source for fermentation, did not show any significant shifts in metabolic pathways.

### Changes in propionate and butyrate production are linked to the substrate but not acetate

**Fig. 4:**
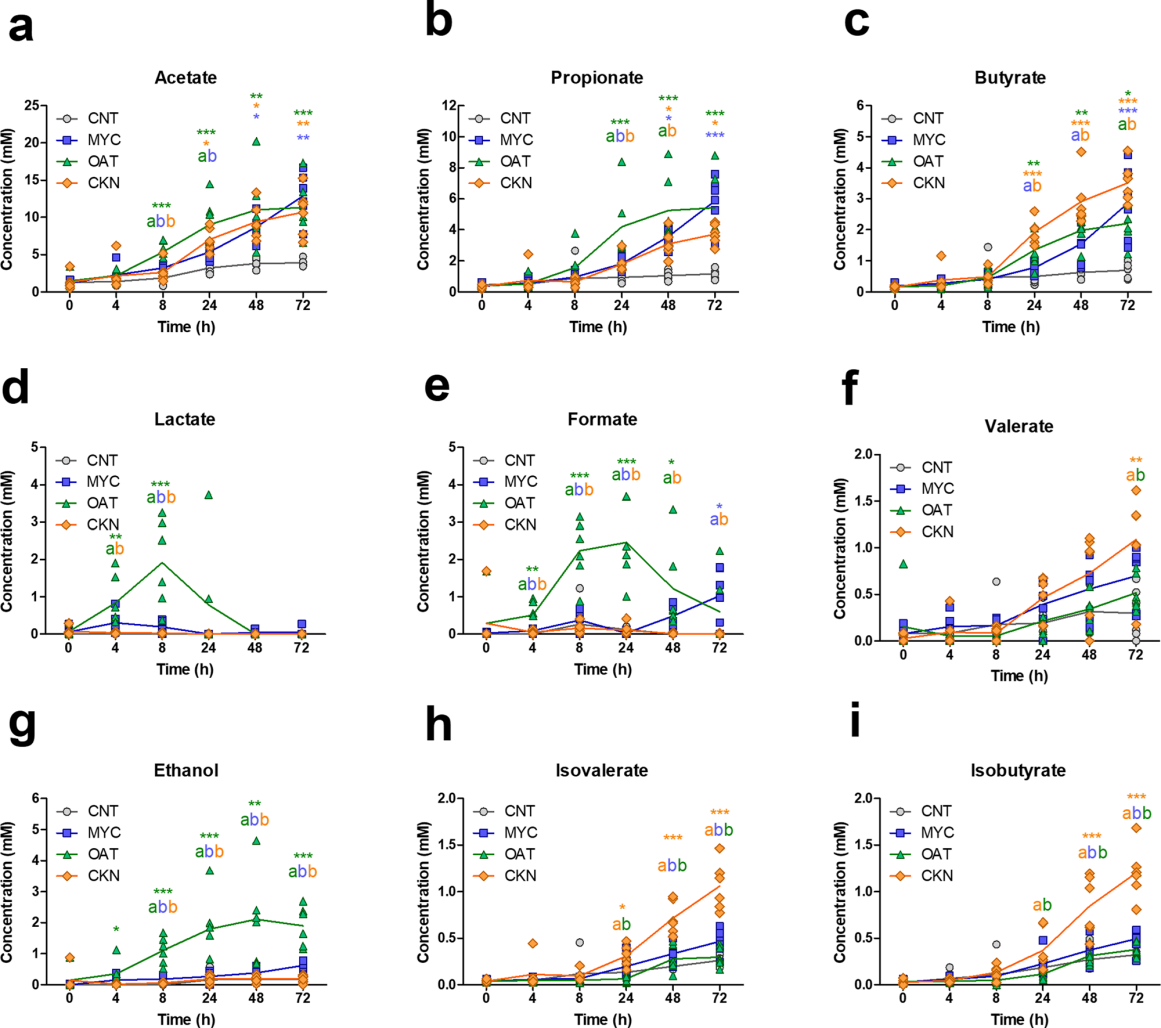
Concentrations of SCFAs, microbial metabolites, and BCFAs measured by NMR from CNT, MYC, OAT, and CKN during 72 h of in vitro colonic fermentation. The concentrations of the following metabolites were measured: **a** Acetate, **b** Propionate, **c** Butyrate, **d** Lactate, **e** Formate, **f** Valerate, **g** Ethanol, **h** Isolvalerate, **i** Isobutyrate. Statistically significant differences in metabolite concentrations were determined by one-way ANOVA, Tukey’s post hoc test (*p* value < 0.05); *p value < 0.05, **p value < 0.01, ***p value < 0.001 are statistically significant compared to the CNT. Letters report statistical differences within the substrates (e.g., MYC vs OAT, MYC vs CKN, OAT vs CKN).

Figure 4 shows the microbial metabolite profiles produced during fermentation. SCFA (A-C), microbial metabolites (D-G) and branched-chain fatty acid (BFCA) (H-I) were measured by NMR from CNT, MYC, OAT, and CKN during 72 h of in vitro colonic fermentation. Acetate (Fig. 4a) was the most abundant for all substrates. Differences were observed in the kinetics of acetate production between the substrates, with OAT producing higher SCFA concentrations than MYC at 8 h and 24 h (*p* value < 0.05) and CKN at 8 h (*p* value < 0.01), although the final concentration was comparable within all the substrates. Similarly, OAT had quicker propionate (Fig. 4b) production kinetics at 24 h than MYC (*p* value < 0.05) and CKN at 24 h and 48 h (*p* value < 0.05). However, the propionate concentration of MYC fermentation increased and was comparable to OAT at 48 h and 72 h, whereas CKN was comparable at the 72 h. Conversely, the butyrate (Fig. 4c) concentration observed for CKN fermentation was higher than MYC at 24 h (*p* value < 0.001) and 48 h (*p* value < 0.01) but comparable at 72 h, whereas it was significantly higher in concentration than OAT at 72 h (*p* value < 0.05). The total SCFA production for CKN was lower than for MYC or OAT, suggesting that CKN either had lower fermentability or was fermented to alternative end products. The CNT sample, lacking a carbon source, showed only low levels of metabolic activity and SCFA production, significantly much lower than any of the other substrates.

### OAT fermentation results in elevated levels of intermediate microbial metabolites

Lactate (Fig. 4d) production was observed in significant amounts only in OAT, increasing at 4 h (*p* value < 0.01) and reaching a peak at 8 h (*p* value < 0.001). No significant changes were observed in MYC and CKN compared to the CNT. Formate (Fig. 4e) had a higher concentration in OAT than MYC and CKN from 4 h to 24 h. However, MYC started increasing formate production from 48 h, whereas the OAT concentration decreased from 24 h, resulting in comparable levels with MYC at 48 h and 72 h. CKN did not show substantial production of formic acid during the whole fermentation. OAT also produced a significantly higher amount of ethanol during fermentation than the other substrates (Fig. 4e), while the production of valerate at 72 h was significantly greater with CKN treatment compared to CNT (*p* value < 0.01) and OAT (*p* value < 0.05), but not statistically significant from MYC. The differences observed in microbial metabolite production reflect differences in fermentation pathways between the substrates relating to their different structures and compositions that may reflect the differences observed in microbial community composition between substrates.

### Colonic fermentation of CKN leads to significantly higher levels of Branched-Chain Fatty Acids (BCFA) than MYC or OAT

The BCFA’s isovalerate (Fig. 4h) and isobutyrate (Fig. 4i) were significantly higher in CKN than MYC and OAT starting from 24 h. The high BCFA in the CKN sample likely reflects the fermentation of protein in the CKN sample that was higher in concentration (~2.90 mg/mL) compared to MYC (~1.5 mg/ml) and OAT (~0.32 mg/mL) (Supplementary Note 3). Despite also being a high source of protein, the MYC did not result in significant production of BCFA, and the BCFA production was similar to the levels seen from OAT.

### Structural changes in MYC matrix after colonic in vitro fermentation

**Fig. 5:**
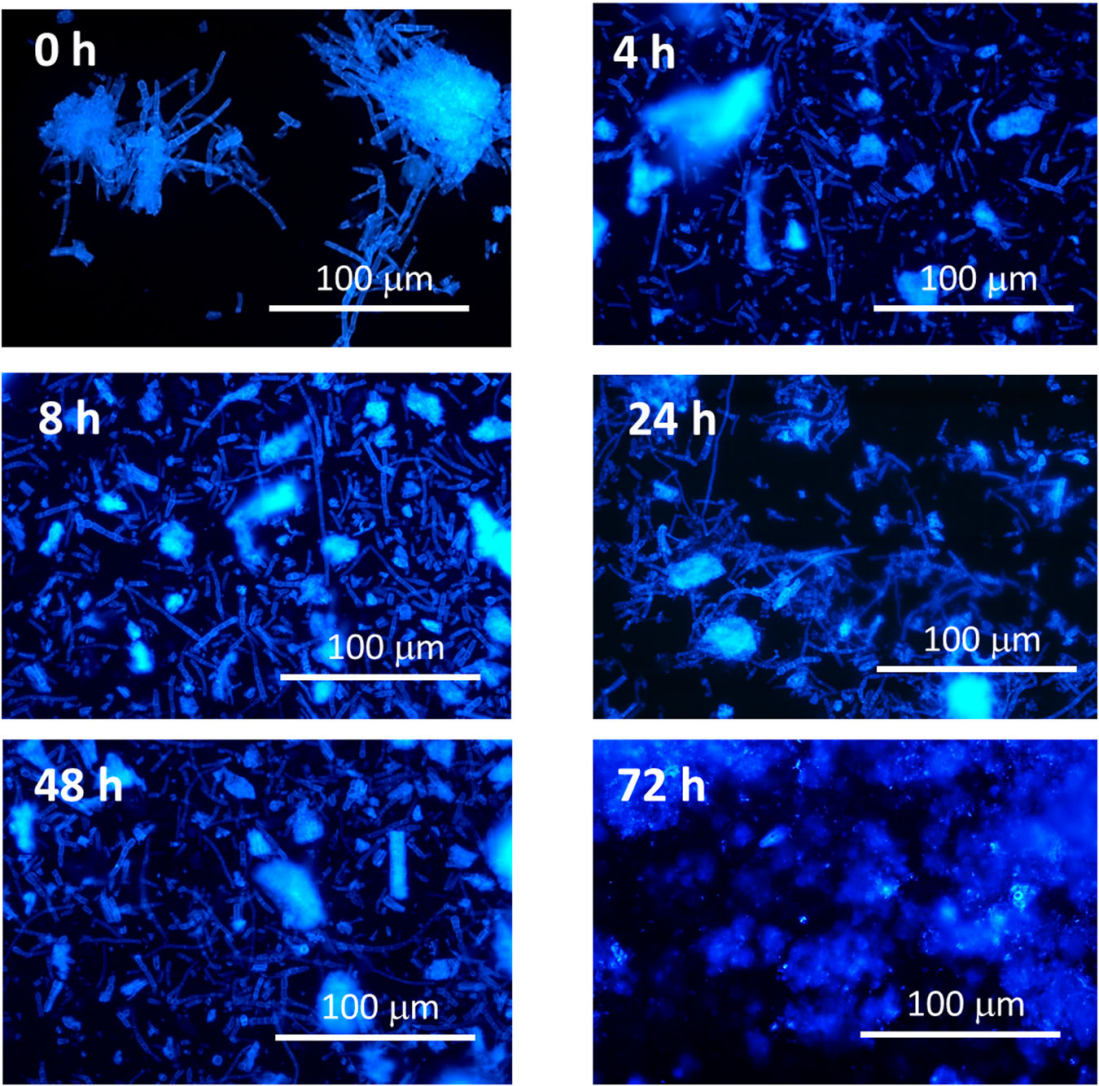
Microscopy of mycoprotein throughout fermentation. Optical microscopy in epifluorescence (with Calcofluor white dye) of MYC at 0, 4, 8, 24, 48, and 72 h of in vitro colonic fermentation.

MYC is composed of filamentous fungal cells and is a complex food ingredient. The filamentous hyphal cells of MYC (tubular structures) were visible at 0 h (Fig. 5). The cells appeared well-defined and intact after the upper GI digestion despite the loss of some nutrients (Supplementary Note 3). The characteristic cellular shape of MYC hyphae was still visible until 48 h, despite being reduced in size as shown by little cellular fragments. However, after 72 h, the cell shape was not observable anymore and appeared as a digested mass. Thus, suggesting that bacteria can ferment the cell wall fibre determining the complex MYC structure and shape, and leading to metabolites production (Fig. 4).

## Discussion

MYC is a novel food derived from the filamentous fungus *F. venenatum* (ATCC PTA-2684). It has been demonstrated that a range of health benefits result from the consumption of MYC, including health effects related to CVD and T2DM^4,6–8,17^. MYC is a source of dietary protein and a potential alternative to meat consumption. Many health benefits have been suggested due to its highly accessible protein content^18^. Unlike meat-based protein sources, MYC is also rich in fibre, primarily mixed linkage β-glucans and chitins, similar to other filamentous fungal cell walls^19^. The fibre in MYC has been demonstrated to be fermentable^12^ and can reduce faecal genotoxins and increase beneficial bacteria in the human gut^20^, but the impact of MYC on microbial metabolite production or microbial community composition, in comparison to other sources of dietary fibre and protein, requires further investigation.

In this study, we have demonstrated consistent increases of three *Bacteroides* species, *B. ovatus, B. xylanisolvens,* and *B. uniformis*, in response to MYC fermentation across stool samples from several volunteers with distinct starting microbiome compositions. *Bacteroides* species are abundant anaerobic bacteria in the human gut microbiota that are recognised as primary degraders of complex carbohydrates such as β-glucans^21–23^. An increase in *Bacteroides* spp. was also observed by Marzorati et al.^24^, following the addition of isolated doses of chitin/glucans (the main components of MYC) to the colonic in vitro model SHIME^®.^ *Bacteroides* species^22^, particularly *B. ovatus*^25^ and *B. uniformis*^26^ have been characterised as primary degraders of β-glucans such as those found in MYC cell walls^19^. These species harbour gene clusters, including GH3, GH158 and GH16 family hydrolases along with glycan-binding proteins, which allow them to break down the linkages in β-(1,3) and β- (1,4) mixed linkage glucans from a wide range of sources, including edible fungi such as MYC^26^. In the present study, these versatile β-glucan degrading species appear to gain a significant advantage and increase in abundance when provided MYC as a substrate, more so even when oat β-glucan is provided in the OAT substrate. The fermentation of OAT results in a distinct microbial community with less enrichment for *Bacteroides* species, and more Bifidogenic effects, highlighting that the microbiome impact of MYC is quite distinct from other beta-glucan-containing fibre sources. *B. ovatus* and *uniformis* are generally regarded as beneficial microbes; for instance, studies on mice have shown that *B. ovatus* can alleviate intestinal inflammation^27^ and *B. uniformis* can decrease hepatic injury and steatosis and improve intestinal dysbiosis and immune dysfunction^28^. Moreover, *B. ovatus* has been described as a novel probiotic that can improve the intestinal environment^29^. Ihekweazu et al.^30^ showed that *B. ovatus* ATCC 8483 monotherapy helps ameliorate colitis and stimulate epithelial recovery in a murine model of inflammatory bowel disease. Similarly, *B. uniformis* may benefit in modulating mice’s metabolic responses by attenuating obesity progression and limiting intestinal absorption of lipids^31^.

Fermentation of MYC fibre results in the production of SCFA’s acetate, butyrate and propionate, which have all been associated with health benefits^32,33^. *Bacteroides* spp. are particularly associated with propionate production^34^ and β-glucans catabolism; in particular, *B. ovatus* has been reported to selectively utilise β-glucans over pectin, xyloglucan and arabinoxylan^22^. Although propionate production is not as rapid for MYC as for OAT, by the end of the fermentation, propionate concentration for both substrates is similar. Lactate was dramatically higher for OAT than MYC and is a precursor for propionate production via the acrylate pathway^35^. This may explain the rapid increase in propionate in OAT from 8 h (Fig. 4b) corresponding with the peak in lactate production, while both lactate production and, subsequently, propionate production occur more slowly in MYC. This suggests that the microbial metabolism of OAT follows different pathways to the MYC, despite β-glucan being the predominant dietary fibre component^36^. The concentration then decreased until it completely disappeared at 48 h. Despite being rich in protein, the BCFA levels produced from MYC fermentation were not significantly different from the control, unlike CKN, which produced much higher quantities of isovalerate and isobutyrate. The dietary fibre present in MYC may be fermented preferentially to protein, as has been demonstrated in animal models^37^. A human intervention comparing MYC with meat demonstrated reduced BCFA following MYC consumption^20^, a finding which has been corroborated in this study demonstrating lower BCFA from MYC fermentation compared to meat, CKN. The intestinal microbiota can produce BCFA through protein fermentation and, particularly, fermentation of branched-chain amino acids (i.e., valine, leucine, and isoleucine)^38^.

In summary, this study showed that MYC and OAT are fermentable substrates promoting a higher differentiation in the colonic microbiota in vitro compared to CKN. *B. uniformis* and *ovatus* species have been found in great abundance in MYC at the end of fermentation (72 h) and are well characterised β-glucan degrading species. Metabolomic analysis revealed the production of SCFAs in all substrates with propionate higher in MYC and OAT, whereas CKN was the only significant producer of BCFA.

In conclusion, this study suggests that in a model system MYC colonic fermentation can promote changes in the colonic microbial profile and has identified bacterial species involved in dietary fibre fermentation (i.e., *B. ovatus, xylanisolvens,* and *uniformis*). Moreover, the production of SCFAs and fermentation metabolites was reported, as well as changes in the MYC matrix after 72 h of colonic fermentation. The disruption of MYC structure was similar to what was observed after incubation with the cell wall-degrading enzyme Driselase™^39^ suggesting that bacteria could digest the cell wall of MYC during simulated colonic fermentation. These findings are relevant to understanding MYC digestion and fermentation, which can help understand how MYC consumption can promote health by improving gut health and gut barrier function.

## Methods

### Chemicals and reagents

All standards and reagents were purchased from Merck (UK) and were ACS grade unless otherwise stated: 4-Hydroxybenzhydrazide (PAHBAH) (H9882, Sigma Aldrich), Calcofluor White stain (18909, Sigma Aldrich), bicinchoninic acid assay kit (BCA) (BCA1), bovine serum albumin used as the BCA standard (P0914), MgSO4·7H2O (Catalogue No. M7506), Pipes buffer (Catalogue No. P6757), NH4Cl (Catalogue No. A9434), Trypticase (Catalogue No. Z699195), MnCl2·4H2O (Catalogue No. M3634), FeSO4·7H2O (Catalogue No. F7002), ZnCl2 (Catalogue No. 208086), CuCl·2H2O (Catalogue No. 224332), CoCl2·6H2O (Catalogue No. C8661), SeO2 (Catalogue No. 200107), NiCl2·6H2O (Catalogue No. 654507), Na2MoO4·2H2O (Catalogue No. M1003), NaVO3 (Catalogue No. 72060), H3BO3 (Catalogue No. B6768), acetic acid (Catalogue No. 71251), propionic acid (Catalogue No. P1386), butyric acid (Catalogue No. B103500), isobutyric acid (Catalogue No. I1754), 2-methylbutyric acid (Catalogue No. 193070), valeric acid (Catalogue No. 240370), isovaleric acid (Catalogue No. 129542), biotin (Catalogue No. B4639), folic acid (Catalogue No. F8758), calcium D-pantothenate (Catalogue No. PHR1232), nicotinamide (Catalogue No. N0636), riboflavin (Catalogue No. R9504), thiamine HCl (Catalogue No. T1270), pyridoxine HCl (Catalogue No. P6280), para-amino benzoic acid (Catalogue No. 579513), cyanocobalamin (Catalogue No. PHR1234), deuterium oxide (Catalogue No. 151882), d4-trimethylsilyl propionic acid sodium salt (Catalogue No. 269913), molecular biology grade water (Catalogue No. W4502), 5 mL Eppendorf^®^ tubes (Catalogue No. Z768744), 1.5 mL LoBind^®^ tubes (Catalogue No. 0030108442), paraformaldehyde (Catalogue No. P6148), MP bio fast DNA spin kit for soil (Catalogue No. 116560200, MP Biomedicals, USA), and Seward Stomacher^®^ Classic Bags (Catalogue No. BA6041/5/500; The Laboratory Store, UK). Megazyme Ltd Mushroom and yeast beta-glucan assay kit (K-YBGL 11/19) was used for MYC, and Megazyme Ltd β-Glucan Assay Kit (Mixed Linkage) (K-BGLU 08/18) was used for OAT (Bray, Ireland).

### Sample preparation

The colonic in vitro fermentation substrates were a control (CNT, faecal slurry without substrate), MYC, OAT, and CKN. Marlow Foods Ltd, UK, provided MYC, whereas OAT (White’s oats, medium cut, UK) and CKN (Iceland Ready Cooked Sliced Chicken Breast, UK) were purchased from a local store. MYC and OAT were hydrated 1:4 with ultra-pure water before digestion. Then, 100 g of the hydrated MYC and OAT samples and 100 g of CKN were transferred into a Duran bottle and cooked for 15 min in a boiling water bath (100 °C). After cooling down at room temperature for 15 min, the samples were digested following the INFOGEST protocol described by Brodkorb et al.^40^ before colonic in vitro fermentation.

### Simulated upper gastrointestinal digestion

After sample preparation, simulated upper GI digestion was carried out as described by Brodkorb et al.^40^ to obtain the substrates for inoculation in the colonic in vitro fermentation experiments.

**Fig. 6:**
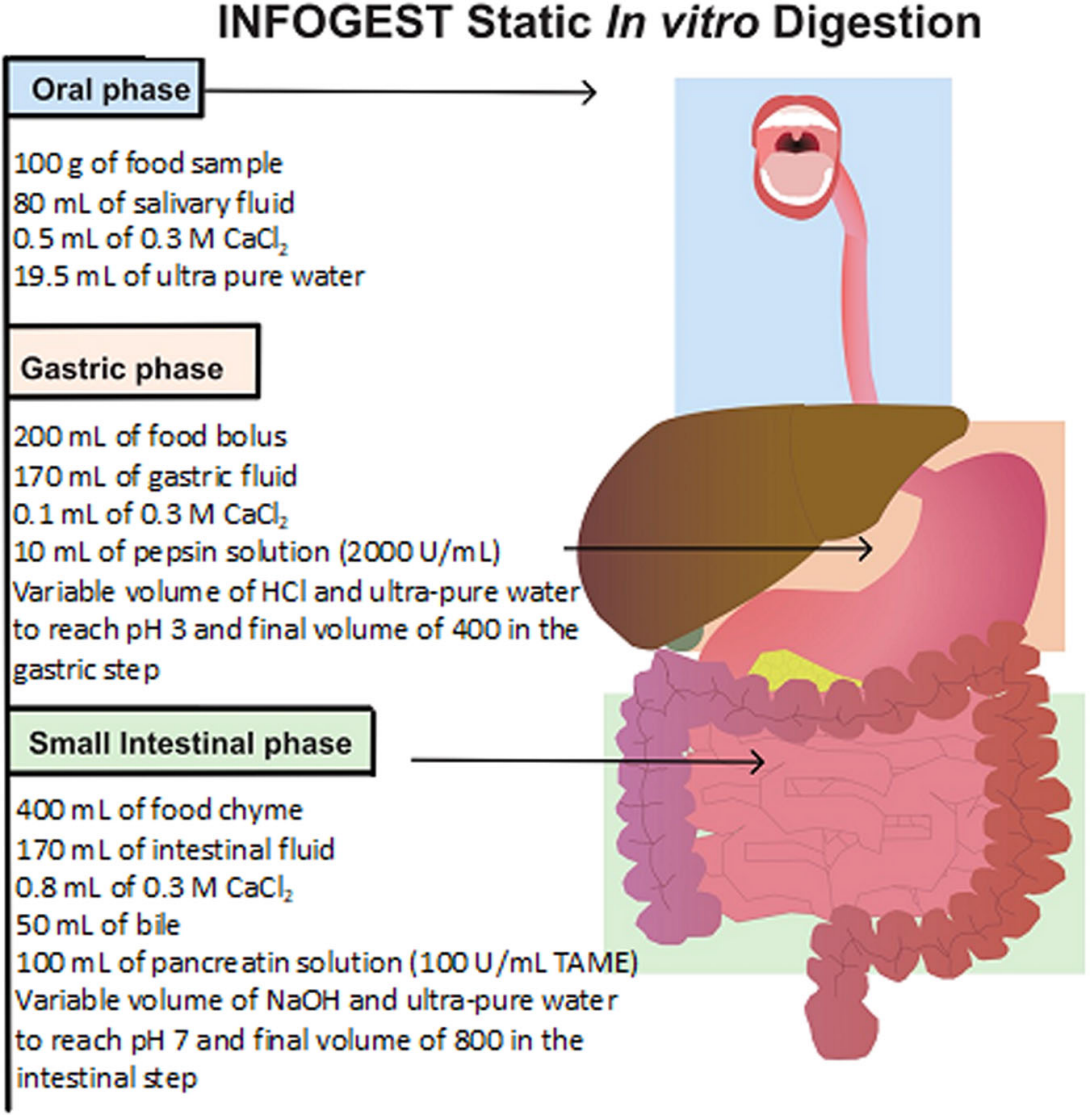
Schematic representation of the INFOGEST static in vitro digestion. Shown with 100 g of starting food and including the oral, gastric, and small intestinal steps. Figure adapted from Brodkorb et al.^40^ Reproduced with permission.

The digestion was carried out with 100 g of food (i.e., 25 g of MYC, OAT, or CKN plus 75 mL of ultra-pure water) with a total digesta volume of 400 mL for the gastric step and 800 mL for the small intestinal phase. A summary of the steps is shown in Fig. 6. At the end of simulated upper GI digestion, the enzyme activity was stopped by adding 5 M NaOH to reach pH 11.0, and steps were performed to obtain the substrates for colonic in vitro fermentation: (1) The supernatant was separated from the pellet by centrifugation at 2800 × *g* for 10 min at room temperature. This procedure was repeated another three times with aqueous ethanol (50% v/v) to wash the pellet from the digestion products (e.g., amino acids, sugars, and free fatty acids). Then, the pellet was freeze-dried (LyoDry Compact, Mechatech, UK) for one week to obtain a dry powder; (2) The supernatant that was separated from step (1) was incubated with aqueous ethanol (80% v/v) for 1 h at room temperature to precipitate the soluble β-glucans in the MYC and OAT samples.

Ethanol precipitation at 70 to 80% in water has been used by numerous studies for carbohydrate precipitation^41^ and to extract plant and fungal β-glucans^42^. The precipitated pellet was centrifuged at 2800 × *g* for 10 min at room temperature, washed three times with ultra-pure water and freeze-dried for one week. The obtained powder was mixed with the dry powder obtained from step (1). Finally, the dry powders from MYC, OAT, and CKN were stored at −20 °C and ready for colonic in vitro fermentation. Results of the protein and carbohydrate digestion in the simulated upper gastrointestinal tract are reported in Supplementary Notes 2 and 3, Table S.4.

### Batch colonic in vitro fermentation

Colonic fermentation was carried out with the method described by Williams et al.^43^ with minor modifications. The media composition and preparation are described in detail in Supplementary Note 1 and Tables S1, S2 and S3. For each substrate, parallel fermentations (in duplicate) were carried out in anaerobic vessels with the media and the faecal slurry produced from the stools of six separate donors. Blanks were established as MYC, OAT, and CKN without the faecal slurry. An aliquot of 5 mL was taken at 0, 4, 8, 24, 48, and 72 h; 2 mL was used for DNA extraction, 1 mL for microscopy, 1 mL for metabolite analysis, and 1 mL was a spare sample.

Ethical approval was granted by the Human Research Governance Committee at the Quadram Institute (IFR01/2015) and London - Westminster Research Ethics Committee (15/LO/2169), and the trial was registered on clinicaltrials.gov (NCT02653001). All ethical regulations relevant to human research participants were followed. Donors fully consented and were healthy adults (≥18 years old), free-living, who had not taken antibiotics in the 3 months prior to donation and were free from gastrointestinal disease. Dietary information was not available for the donors. Signed informed consent was obtained from the participants before donation. The stool sample was collected by the participant, stored in a closed container under ambient conditions, transferred to the laboratory and prepared for inoculation within 2 h of excretion.

### Substrates and faecal slurry preparation

The day before the experiment, the substrate (0.5 g) was added to 76 mL of basal solution, 5 mL of vitamin/sodium-carbonate solution, and 1 mL of reducing solution (Supplementary Note 1). The bottles were bubbled for 4 min with CO2 to make the system anaerobic, which was indicated by the decolourisation of the resazurin prior to faecal inoculation and incubated at 37 °C overnight to allow the substrates to hydrate.

The stool sample was transferred to a stomacher bag and prepared at a 1:10 ratio of the stool sample to sterile anaerobic phosphate-buffered saline (PBS). The Seward Stomacher^®^ bag containing the slurry was added to a stomacher machine (Seward™ 030010159) and mixed for 30 s. Using a 19 G needle and syringe, 3 mL of the slurry was injected into the serum bottle to start the fermentation process. Aliquots of 5 mL were taken with a 19 G syringe at 0, 4, 8, 24, 48, and 72 h and stored at −80 °C for DNA and metabolomic analysis. In contrast, samples for microscopy were fixed on the same day of the experiment.

### Metagenomic analysis

DNA extraction was carried out per the manufacturer’s instructions with the MP Bio fast DNA spin kit for soil (MP Biomedical, Solon, USA).

Sample aliquots from the colonic in vitro fermentation were pelleted by centrifugation (2800 × *g* for 10 min at 4 °C) and frozen (−80 °C). Into frozen sample pellet, 400 µL of water (molecular biology grade) was added for the 0, 4, 8 h timepoints, whereas 500 µL of water was added for the 24, 48, 72 h timepoints. This pellet slurry (500 µL) was transferred to a Lysing Matrix E tube where980 µL of sodium phosphate buffer and 120 µL of MT buffer were added. The samples were homogenised in the FastPrep24 bead-beating instrument (MP Biomedicals, Solon USA) for 3 min (3 runs of 60 s each, with 5 min rest on ice in between). Afterwards, samples were centrifuged at 14,000 × *g* for 15 min, and the sample supernatant was transferred into clean 2 mL microcentrifuge tubes with 250 µL of protein precipitate solution. The tubes were mixed by shaking by hand 10 times before centrifugation at 14,000 × *g* for a further 10 min. The supernatant was transferred to 5 mL tubes with a resuspended binding matrix (1 mL of silica slurry that binds DNA from lysates), and the tubes were inverted for 2 min allowing the DNA to bind. The tubes were placed in a rack and allowed to settle for 1 h. Then, 500 µL of the supernatant was removed, and the binding matrix was resuspended in the remaining supernatant. The washing steps involved the transfer of 700 µL of the mixture to a SPIN filter centrifuged at 1000 × *g* for 2 min to empty the catch tube and transfer the leftover mix. This process was repeated until all the mixture had been added to the SPIN filter. The SPIN filter was washed with 500 µL of ethanol-based wash solution SEWS-M. The samples were centrifuged at 14,000 × *g* for 5 min, and the catch tube was emptied. Then, samples were centrifuged a second time (Dry spin) at 14,000 × *g* for 5 min to remove the residual wash solution. The catch tube was replaced with a clean tube (1.5 mL LoBind Eppendorf^®^ tubes), and samples in the SPIN filter were air-dried for 10 min at room temperature and 5 min at 37 °C. Then, 65 µL of DNA extraction solution was added to the Binding Matrix and incubated at room temperature for 5 min before being centrifuged at 6600 × *g* for 2 min with lids open to bring eluted DNA into the tube. The SPIN Filter was discarded, and the eluted DNA was stored at −20 °C.

### Library preparation

Genomic DNA was normalised to 5 ng/µl with 10 mM Tris-HCl buffer. A miniaturised reaction was set up using the KAPA UDI Primer Mixes, 1–96 (Roche Catalogue No 09134336001). A master mix was prepared from 0.5 µL tagmentation buffer 1, 5 µL bead-linked transposomes, and 4 µL PCR-grade water. Then, 5 μL of this master mix was added to a chilled 96-well plate. Afterwards, 2 µL of normalised DNA (10 ng total) was mixed with the 5 µL tagmentation mix and heated to 55 °C for 15 min in a PCR block. A PCR master mix was made up from the Kap2G Robust PCR kit (Sigma Catalogue No. KK5005) by using 4 µL kapa2G buffer, 0.4 µL dNTP, 0.08 µL Polymerase and 3.52 µL PCR-grade water, and 8 µL were taken and added to a 96-well plate before adding 5 µL of UDI index primers. Finally, the 7 µL of Tagmentation mix was added and mixed. The PCR was run at 72 °C for 3 min, 95 °C for 1 min, 14 cycles of 95 °C for 10 s, 55 °C for 20 s and 72 °C for 3 min. Following the PCR reaction, the libraries were quantified using the Quant-iT dsDNA Assay Kit, high sensitivity kit (Catalogue No. 10164582) analysed using a FLUOstar Optima plate reader. Libraries were pooled following quantification in equal quantities. The final pool was double-SPRI size selected between 0.5 and 0.7× bead volumes using KAPA Pure Beads (Roche Catalogue no. 07983298001). The final pool was quantified on a Qubit 3.0 instrument and analysed on a D5000 ScreenTape (Agilent Catalogue No. 5067–5588 & 5067–5589) using the Agilent Tapestation 4200 to calculate the final library pool molarity. qPCR was carried out on an Applied Biosystems StepOne Plus machine. Samples to be quantified were diluted 1 in 10,000. A PCR master mix was made up using 10 μL KAPA SYBR FAST qPCR Master Mix (2×) (Sigma Catalogue No. KK4600), 0.4 μL ROX High, 0.4 μL 10 μM forward primer, 0.4 μL 10 μM reverse primer, 4 μL template DNA, and 4.8 μL PCR-grade water. The PCR programme run was 95 °C for 3 min, 40 cycles of 95 °C for 10 s, and 60 °C for 30 s. Standards were made from a 10 nM stock of Phix, dilution made in PCR-grade water; the standard range was from 20 pmol to 0.0002 pmol. Samples were then sent for shotgun metagenomic sequencing to be run along with sample names and index combinations used.

### Shotgun metagenomics sequencing

Shotgun metagenomic sequencing was performed by Genewiz European Headquarters (Germany), using Illumina NovaSeq to generate 150 bp paired-end libraries to a sequencing depth of ~10 million reads per sample. The demultiplexed FASTQ files were used for the bioinformatics analysis. The data have been deposited with links to the BioProject accession number PRJNA928249 in the NCBI BioProject Database^44^.

### Metagenomics read processing

The analysis of the metagenomics data was performed using the bioBakery suite^45^, which contains computational methods for quality control and contaminant depletion (KneadData), taxonomic profiling (MetaPhlAn), functional profiling (HUMAnN) and multivariate association analysis (MaAsLin2). The analysis is performed to investigate the effect of the different substrates on the microbial and functional profiles over time.

First, raw sequencing reads are processed to remove host contaminants, and bacterial rRNA reads using KneadData (Supplementary note 5, Figure S2)^46^. KneadData was used with three databases: (a) the human (hg37) reference database (which includes the human genome and transcriptome, based on the Decoy Genome^47^; (b) a contaminant database taken from Breitwieser et al.^48^; and (c) a bacterial rRNA reads from SILVA database^49^. In addition, KneadData also performs quality control using Trimmomatic^50^, removing adaptor sequences, bases with low quality and short reads, as well as trimming overrepresented sequences (using parameter –run-trim-repetitive). High-quality, trimmed and non-human reads are used for downstream analyses.

### Microbial community profiling

Non-host and high-quality reads were used to estimate taxonomic profiles for each sample. MethaPhlAn4.0.4 was used to identify the microbes and their abundances from metagenomics reads by mapping them to a database of unique clade-specific marker genes. We used the ChocoPhlAn database, vJan21_CHOCOPhlAnSGB_202103, which contains marker genes from over ~1 M microbial genomes^45,51^.

Comparison of the taxonomic profiles for each sample was initially explored using a PCoA plot. PCoA plots were generated using the R package vegan (2.6-2) with Bray–Curtis distance and plotted using the Phylosmith package^52^. The community composition of the substrates at the 72 h timepoint was compared using a PERMANOVA test with the adonis2 function of the vegan (version 2.6-4) software package^53^.

### Functional analysis

Functional analysis was performed using HUMAnN v3.6.0, which is the HMP (Human Microbiome Project) Unified Metabolic Analysis Network method for profiling the abundance of microbial metabolic pathways and other molecular functions from metagenomic sequencing data. HUMAnN infers community function directly from short DNA reads^45,54^. It uses KEGG orthology to estimate abundances for each orthologous gene family (a group of genes that perform the same biological roles) in the community. The analysis with HUMAnN3 was done using a nucleotide mapping and translated search to provide organism-specific gene and pathways abundance profiles from our high-quality shotgun metagenomics reads. Gene families are annotated using UniRef90 definitions^55^. Pathways are annotated using MetaCyc definitions^56,57^. For this analysis, HUMAnN3 was used with the default parameters and uniref90 to annotate gene families. Relative abundances were transformed to CPM (copies per million) units to account for sample sequencing depth to compare profiles across samples.

### Statistical associations with a linear model (MaAsLin2)

MaAsLin2^58^ was used to find associations between pathway relative abundances and the effect of each substrate over time. We use MaAsLin2 on CPM values, with a Linear Mixed-Effect Model (LM), TSS normalisation, log transformation and a minimum prevalence of 0.05. Corrected significant p-values were estimated using the Benjamini-Hochberg, BH, correction method. The following formula is used for the Linear Mixed-Effect Model, where the relative abundance of the pathway is determined by the interaction between the substrate (CKN, MYC or OAT) and timepoint (0, 4, 8, 24, 48, and 72 h):

$${{{{{\rm{expression}}}}}} \sim {{{{{\rm{substrate}}}}}}+{{{{{\rm{time\; point}}}}}}$$

In addition, we also consider the donor as a grouping variable to represent the grouping effect when samples are taken over time for the same donor:

$${{{{{\rm{random}}}}}}\_{{{{{\rm{effect}}}}}} \sim {{{{{\rm{donor}}}}}}$$

### Metabolomic analysis

NMR spectra were acquired on a Bruker Avance Neo 600 MHz spectrometer equipped with a 5 mm TCI cryoprobe and a 24-position SampleCase autosampler. The experiments were carried out at 298 K in saline phosphate buffer (1.9 mM Na2HPO4, 8.1 mM NaH2PO4, 150 mM NaCl, and 1 mM TSP (sodium 3-(trimethylsilyl)-propionate-d4)) in D_2_O (deuterium oxide)^59^. 400 µL of the sample was mixed with 200 µL of buffer prior to loading into the NMR tube. TopSpin software (version 4.1.1, Bruker, USA) was used for data acquisition and processing.

NMR spectra with water presaturation during relaxation delay were acquired using a standard pulse sequence noesygppr1d, which suppressed residual water by applying low power selective irradiation at the water frequency during the recycle delay (D1 = 4 s) and mixing time (D8 = 0.010 s). Four dummy scans were used for equilibration, followed by 64 scans (acquisition time = 2.6 s) collected into 65,536 points of NMR spectra with 20.8 ppm width. The spectra were processed using the Bruker AMIX data processing package. The Free Induction Decays were zero-filled, and an exponential 0.3 Hz line-broadening function was applied before the Fourier transformation. All NMR spectra were automatically phased, and a baseline correction was applied. The TSP peak was assigned at δ 0.00 ppm for an internal chemical shift reference. The determination and quantification of the peaks in the spectra corresponding to specific compounds were carried out with Chenomx version 8 (Canada).

### Microscopy analysis

The samples were fixed with paraformaldehyde (PFA). Briefly, 1 mL taken from the respective timepoint was centrifuged at 13,000 × *g* for 3 min to remove the supernatant and resuspended in 400 mL of PBS and 1.1 mL of PFA solution (4% in PBS, pH 6.9) at 4  °C overnight. Then, the tubes were centrifuged at 13,000 × *g* for 3 min to remove the supernatant, and a further three washes were performed with 1 mL PBS. Finally, the pellet was resuspended in 600 µL of ethanol (50% v/v) in PBS and stored at −20 °C. Optical microscopy was performed through an Olympus, Japan, BX60 optical microscope; micrographs were acquired with a Jenoptik ProgRes C10plus camera (Jenoptik AG, Germany). After fixation, the sample pellet was analysed in epifluorescence mode, and Calcofluor white dye was used in proportion 2:1 (dye:samples).

### Statistics and reproducibility

Data were analysed by GraphPad Prism version 5 for Windows (GraphPad Software, USA). Values were expressed as average ± confidence interval at 95% (CI95%) of six independent measures (*n* = 6) for the metabolomic analysis and standard deviation (SD) of six independent measures (*n* = 6) for protein and carbohydrate analysis (Supplementary Note 3). Statistical significance was set at *p* value < 0.05 using one-way ANOVA with Tukey’s post-hoc method. For SCFAs analysis, the significance was examined between 1) the control group (CNT) vs the treatment groups (MYC, OAT, and CKN) and 2) treatment group vs treatment group. The MDS plots were generated using the metaMDS function of the R package vegan, version 2.5–7 (R Studio, USA).

### Reporting summary

Further information on research design is available in the Nature Portfolio Reporting Summary linked to this article.

### Supplementary information

Supplementary Information

Description of Additional Supplementary Files

Supplementary Data 1–5

Reporting Summary

## Data Availability

Raw sequencing data and associated metadata (participant details, substrate and sampling timepoint) can be accessed through NCBI SRA project number PRJNA928249. The source data underlying Figs. 1 and 2 can be found in Supplementary Data 1 and 2, the source data underlying Fig. 3 can be found in Supplementary Data 3 and 4, and the source data underlying Fig. 4 can be found in Supplementary Data 5.
