# Supplementary material for: Colonic in vitro fermentation of mycoprotein promotes shifts in gut microbiota, with enrichment of Bacteroides species
Source: Commun Biol. 2024 Mar 5;7:272. doi: 10.1038/s42003-024-05893-4 (PMC10915147; doi:10.1038/s42003-024-05893-4)
Supplement: Supplementary file 1 — Supplementary Information [file 42003_2024_5893_MOESM1_ESM.pdf]

Supplementary Material

Supplementary Note 1. Media Preparation

The media was composed of 76 mL of basal solution, 5 mL vitamin/bicarbonate solution, and 1 mL reducing agent. The preparation of the individual solutions is described as follows. The basal solution was made by adding the buffer salts of Table S.1 in a 2 L beaker.

Table S.1. List of mineral salts used for the trace mineral solution prepared in 0.02 M HCl, made up to 500 mL of ultra-pure water.

Chemical	Quantity (g) for 2 L
KCl	1.42
NaCl	1.42
$CaCl_2 \cdot 2H_2O$	0.47
$MgSO_4 \cdot 7H_2O$	1.19
Pipes buffer	3.56
NH_4Cl	1.28
Trypticase	2.37

Then, 2.35 mL of resazurin (0.05 g in 50 mL of ultra-pure water) and 23.78 mL of haemin solution (0.05 g in 25 mL of 0.05 M NaOH, made up to 500 mL using boiling ultra-pure water) were added. This was followed by adding 23.78 mL of trace mineral solution and 23.78 mL of fatty acid solution (Table S.2).

Table S.2. List of mineral salts (left) used for the trace mineral solution prepared in 0.02 M HCl, made up to 500 mL of ultra-pure water, and fatty acids (right) used for the fatty acid solution prepared in 200 mL of 0.2 M NaOH.

Mineral Salts		Fatty Acids	
Chemical	Quantity (mg)	Chemical	Quantity (mL) for 200 mL
$MnCl_2·4H_2O$	12.5	Acetic acid	1.37
$FeSO_4·7H_2O$	10	Propionic acid	0.60
$ZnCl_2$	12.5	Butyric acid	0.37
$CuCl·2H_2O$	12.5	Isobutyric acid	0.09
$CoCl_2·6H_2O$	25	2-Methylbutyric acid	0.11
SeO_2	25	Valeric acid	0.11
$NiCl_2·6H_2O$	125	Isovaleric acid	0.11
$Na_2MoO_4·2H_2O$	125		
$NaVO_3$	15.7		
H_3BO_3	125		

The volume was made up to 2 L, and the pH was adjusted to 6.8 with KOH (4 M). The solution was then bubbled with CO_2 overnight, and 76 mL of basal solution was dispensed into 100 mL serum bottles, capped, and autoclaved at 121 °C for 15 min. Once the basal solution was at room temperature, the batches were stored at 4 °C.

Table S.3 shows the preparation of the vitamin solution. The carbonate solution was prepared by adding 4.1 g of Na_2CO_3 to 500 mL of boiled ultra-pure water and bubbling with CO_2 for 30 min (until it reached room temperature). Then, 60 mL was transferred into serum bottles, capped, crimped, and autoclaved at 121 °C for 15 min. Afterwards, 15 mL of vitamin solution was added to the 60 mL bottles containing sodium carbonate.

Table S.3. List of vitamins used for the vitamin solution prepared in 500 mL with 27.35 g KH_2PO_4.

Chemical	Quantity (mg) for 500 mL
Biotin	10
Folic acid	10
Calcium D-pantothenate	8
Nicotinamide	32
Riboflavin	82
Thiamine HCL	82
Pyridoxine HCl	82
P*ara*-amino benzoic acid	10.2
Cyanocobalamin (vitamin B12)	10.3

The reducing solution was prepared with 1 g of L-Cysteine HCl and 1 g of $Na_2S{\cdot}9H_2O$ in boiling ultra-pure water, made up to 50 mL at pH 10.0 (using 4 M NaOH).

Supplementary Note 2. Protein and Reducing Sugar Analysis & β-glucans measurement

At the end of the simulated GI digestion, protein and reducing sugar analysis was carried out on the supernatant of the digested samples. The bicinchoninic acid assay (BCA) and p-hydroxybenzoic acid hydrazide (PAHBAH) assays were used for protein and reducing sugar estimation, respectively.

Briefly, for BCA, 10 µL of standard (BSA) or unknown samples was incubated with 200 µL of working reagent of the BCA assay kit for 30 min at 37 °C. Absorbance was then measured at 562 nm by Benchmark Plus™ spectrophotometer (Bio-Rad, UK) in a 96-well microplate (1). For PAHBAH, 100

μL of each standard or sample was mixed with 1 mL of PAHBAH solution in an Eppendorf® Safe-lock tube that was boiled in a water bath for 5 min. The tubes were allowed to cool at room temperature, and the absorbance was then measured at 405 nm by Benchmark Plus™ UV/Vis spectrophotometer in a 96-well microplate (2). The β-glucan content of MYC and OAT was measured as described in R. Colosimo et al. (3), with Megazyme kits, from the pellet obtained by mixing the recovered pellet from the aqueous ethanol (80% v/v) precipitation and the washed pellet after simulated GI digestion.

The values (%wt) obtained from BCA, PAHBAH, and β-glucan content analysis were expressed as residual proteins, carbohydrates (starch/α-glucans for OAT and glycogen/α-glucans for MYC), and β-glucans in the sample pellet after simulated upper GI digestion and before *in vitro* colonic fermentation. This was done by subtracting the value obtained by BCA, PAHBAH and β-glucan content analysis from the total starting value (100%wt) of each sample protein, carbohydrates, and β-glucans before digestion.

Supplementary Note 3. Protein and Carbohydrate Digestion in the Upper Gastrointestinal Tract

The samples were subjected to INFOGEST simulation of the upper gastrointestinal tract before *in vitro* colonic fermentation. The undigested protein, reducing sugars and β-glucans that remained in the samples pellet after simulated GI digestion were analysed and are reported in Table S.4.

Table S.4. Total protein, reducing sugars and β-glucans from the whole respective sample (wt%) remaining in the substrates pellet (MYC, OAT, and CKN) after simulated upper GI digestion and before inoculation in the colonic batch fermentation. n/d: not detected; n/a: not applicable.

Sample	Proteins (wt%)	Starch/α-glucans (OAT), Glycogen α-glucans (MYC) (wt%)	β–Glucans (wt%)
**MYC**	68.13 ± 0.86	30.32 ± 3.75	72.78 ± 6.30
**OAT**	74.64 ± 1.04	24.95 ± 1.16	81.92 ± 3.11
**CKN**	41.63 ± 2.97	n/d	n/a

MYC had 68.13 ± 0.86 wt% of protein (29.98 ± 0.37 g/100g dry weight in the pellet after simulated upper GI digestion and ~1.50 mg/mL in the colonic batch (0.5 g of MYC in 100 mL of media)) and 30.32 ± 3.75 wt% of carbohydrates (3.64 ± 0.45 g/100g dry weight in the pellet after simulated upper GI digestion and ~0.16 mg/mL in the colonic batch) remaining in the pellet after simulated upper GI digestion. These measures suggest that ~32 wt% and ~70 wt% of MYC proteins and carbohydrates, respectively, were released from the pellet (in the supernatant) and digested. This is similar to previously determined protein release values for MYC following *in vitro* digestion (4). Similarly, OAT retained 74.64 ± 1.04 wt% of protein (6.42 ± 0.09 g/100g dry weight in the pellet after simulated upper GI digestion and ~0.32 g in the colonic batch (0.5 g of OAT in 100 mL of media)) and 24.95 ± 1.16 wt% of carbohydrates in the pellet after upper simulated GI digestion (13.02 ± 0.61 g/100g dry weight in the pellet after simulated upper GI digestion and ~2.60 g in the colonic batch). This suggests that ~25 wt% of its protein and ~75 wt% of its carbohydrates were released. On the other hand, CKN carbohydrates were not detected, whereas the protein remaining value was 41.63 ± 2.97 wt% (40.66 ± 2.89 g/100g dry weight in the pellet after simulated upper GI digestion and ~2.03 g in the colonic batch (0.5 g of CKN

in 100 mL of media)), suggesting a protein release of ~59 wt%. The protein concentration in MYC (~1.50 mg/mL) was lower compared to CKN (~2.03 mg/mL) during *in vitro* colonic fermentation, thus, potentially leading to a higher BCFA production from the CKN substrate compared to MYC (Figure 4. H, I).

The pellets from MYC and OAT obtained after simulated gastrointestinal digestion were mixed with the respective pellet fraction recovered from the aqueous ethanol (80% v/v) precipitation applied to recover soluble β-glucans from the supernatant after digestion (~~as described in Section 2.2.1).~~ The β-glucans value was 72.78 ± 6.30 wt% (13.10 ± 1.13 g/100g dry weight in the pellet after simulated upper GI digestion and ~0.66 mg/mL in the colonic batch (0.5 g of MYC in 100 mL of media)) for MYC and 81.92 ± 3.11 wt% (6.82 ± 0.26 g/100g dry weight in the pellet after simulated upper GI digestion and ~0.34 mg/mL in the colonic batch (0.5 g of OAT in 100 mL of media) for OAT. These measurements imply that ~27 wt% of the MYC and ~18 wt% of the OAT β-glucans were lost after the upper GI simulated digestion, despite the ethanol precipitation that aimed to recover the soluble β-glucans released in the supernatant. This loss can be due to the release of soluble low molecular-weight β-glucans from the cell walls that are not precipitated by ethanol (5).

Supplementary Note 4. Bacteroidetes/Firmicutes ratio

Figure S.1 shows the Bacteroidetes/Firmicutes ratio for each of the substrates at each times point.

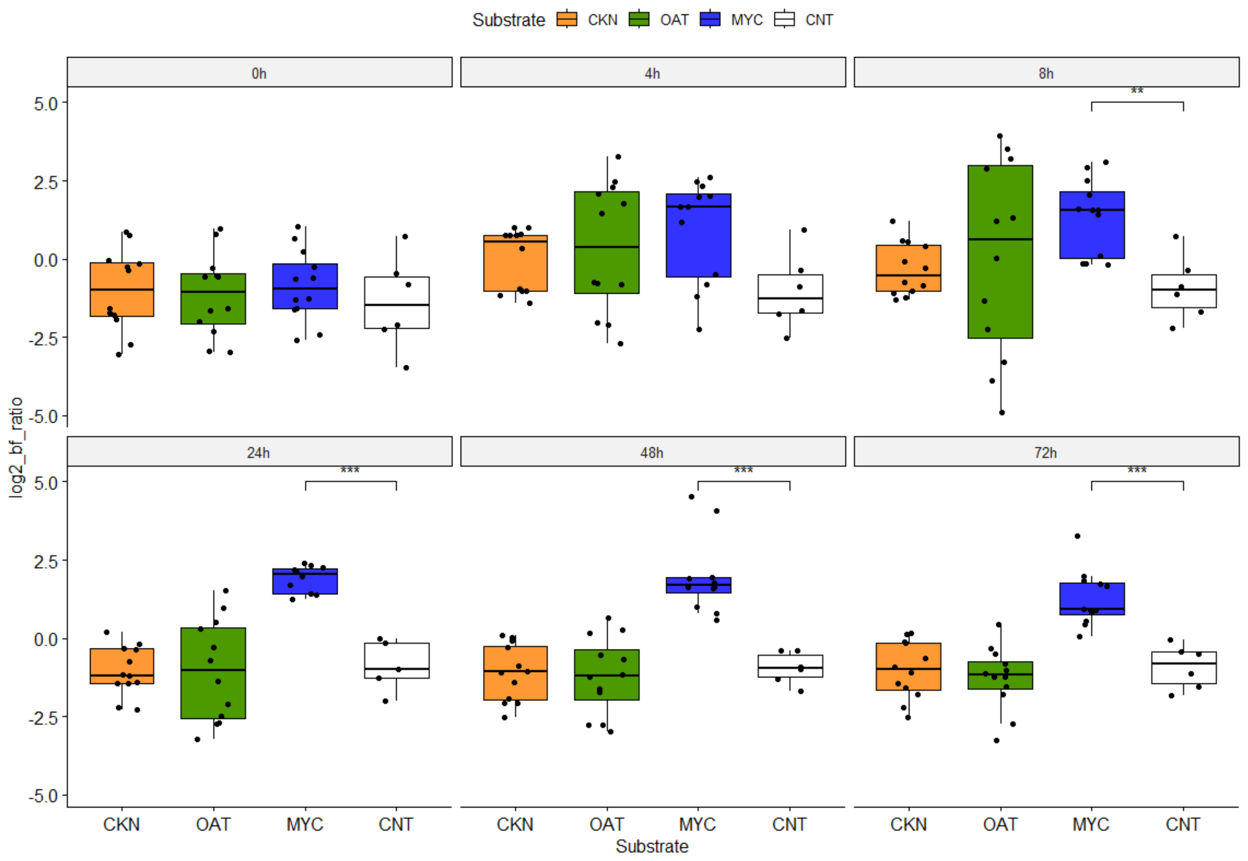

Figure S.1 Changes in microbial community Bacteroidetes/Firmicutes ratio for CNT, MYC, OAT and CKN grouped for time points (0, 4, 8, 24, 48, 72 h). Statistical significance testing was carried out with a Wilcoxon test relative to the CNT sample and p-values were corrected for multiple-comparisons with the Benjamini-Hochberg method. * p-value <= 0.05, ** p-value <= 0.01, *** p-value <= 0.001.

Supplementary Note 5. Processing Metagenomics Data Quality Control

Figure S.2 shows the average number of reads used for downstream analysis for each substrate.

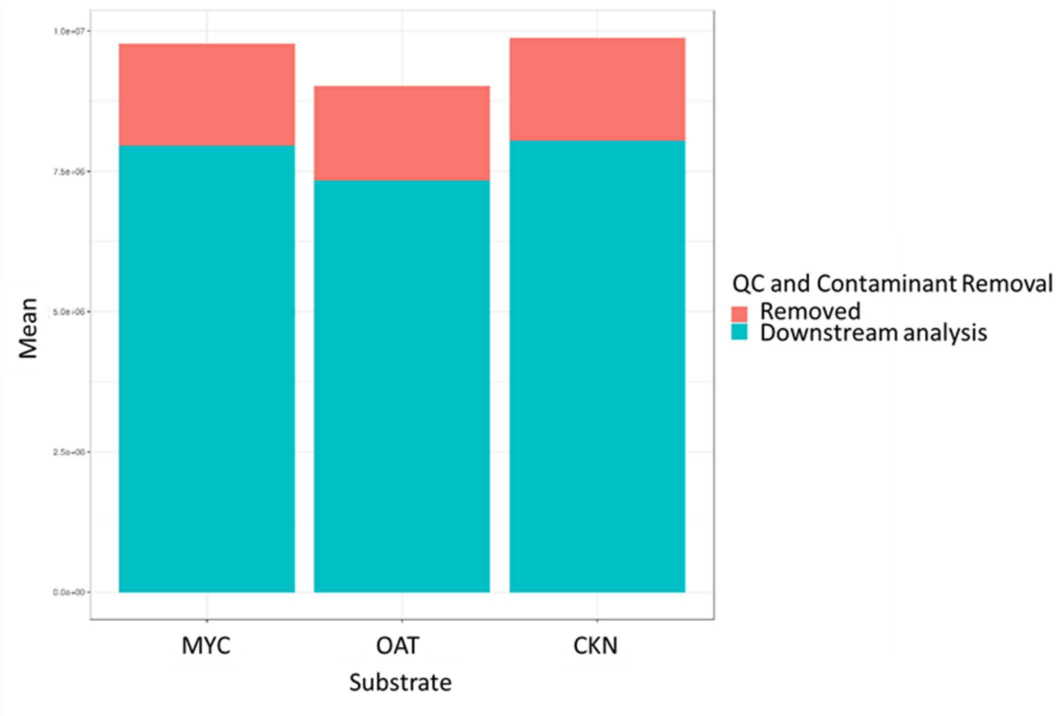

Figure S.2 Quality control and filtering contaminants using KneadData (6). The plot shows the mean number of reads removed for samples treated with each substrate and the number of reads retained for downstream analysis. KneadData was used for quality control (trim adaptor sequences, remove low-quality and short reads) and remove contaminant reads and repetitive sequences.

Supplementary References

1. Smith PK, Krohn RI, Hermanson GT, Mallia AK, Gartner FH, Provenzano MD, Fujimoto EK, Goeke NM, Olson BJ, Klenk DC. 1985. Measurement of protein using bicinchoninic acid. Analytical Biochemistry 150:76-85.

2. Lever M. 1972. A new reaction for colorimetric determination of carbohydrates. Analytical Biochemistry 47:273-279.
3. Colosimo R, Mulet-Cabero A-I, Cross KL, Haider K, Edwards CH, Warren FJ, Finnigan TJA, Wilde PJ. 2021. β-glucan release from fungal and plant cell walls after simulated gastrointestinal digestion. Journal of Functional Foods 83:104543.
4. Colosimo R, Warren FJ, Finnigan TJA, Wilde PJ. 2020. Protein bioaccessibility from mycoprotein hyphal structure: In vitro investigation of underlying mechanisms. Food Chemistry 330:127252.
5. Manzi P, Pizzoferrato L. 2000. Beta-glucans in edible mushrooms. Food Chem 68:315-318.
6. Biobakery/KneadData. Accessed: 10/11/2021. Quality control tool on metagenomic and metatranscriptomic sequencing data. URL: https://github.com/biobakery/kneaddata. Accessed
