# Supplementary material for: Colonic in vitro fermentation of mycoprotein promotes shifts in gut microbiota, with enrichment of Bacteroides species
Source: Commun Biol. 2024 Mar 5;7:272. doi: 10.1038/s42003-024-05893-4 (PMC10915147; doi:10.1038/s42003-024-05893-4)
Supplement: Supplementary file 2 — Description of Additional Supplementary Files [file 42003_2024_5893_MOESM2_ESM.pdf]

Description of Additional Supplementary Files

File name: Supplementary Data 1
Description: MetaPhlAn4 taxonomic profiles for all samples.

File name: Supplementary Data 2
Description: Merged taxonomic profiles averaged by substrate/timepoint.

File name: Supplementary Data 3
Description: Metabolic gene pathways normalized to copies per million (CPM) using HuMAnN3 tool.

File name: Supplementary Data 4
Description: Output of MaAsLin2 modelling of interactions between metabolic pathways, time and substrate.

File name: Supplementary Data 5
Description: Metabolite concentrations determined by 1H NMR.
