# Supplementary material for: Colonic in vitro fermentation of mycoprotein promotes shifts in gut microbiota, with enrichment of Bacteroides species
Source: Commun Biol. 2024 Mar 5;7:272. doi: 10.1038/s42003-024-05893-4 (PMC10915147; doi:10.1038/s42003-024-05893-4)
Supplement: Supplementary file 4 — Reporting Summary [file 42003_2024_5893_MOESM4_ESM.pdf]

nature portfolio

Corresponding author(s): Frederick Warren

Last updated by author(s): Jun 1, 2023

Reporting Summary

Nature Portfolio wishes to improve the reproducibility of the work that we publish. This form provides structure for consistency and transparency in reporting. For further information on Nature Portfolio policies, see our Editorial Policies and the Editorial Policy Checklist.

Statistics

For all statistical analyses, confirm that the following items are present in the figure legend, table legend, main text, or Methods section.

n/a	Confirmed	
☐	☒	The exact sample size (*n*) for each experimental group/condition, given as a discrete number and unit of measurement
☐	☒	A statement on whether measurements were taken from distinct samples or whether the same sample was measured repeatedly
☐	☒	The statistical test(s) used AND whether they are one- or two-sided<br>*Only common tests should be described solely by name; describe more complex techniques in the Methods section.*
☐	☒	A description of all covariates tested
☐	☒	A description of any assumptions or corrections, such as tests of normality and adjustment for multiple comparisons
☐	☒	A full description of the statistical parameters including central tendency (e.g. means) or other basic estimates (e.g. regression coefficient) AND variation (e.g. standard deviation) or associated estimates of uncertainty (e.g. confidence intervals)
☐	☒	For null hypothesis testing, the test statistic (e.g. *F*, *t*, *r*) with confidence intervals, effect sizes, degrees of freedom and *P* value noted<br>*Give P values as exact values whenever suitable.*
☒	☐	For Bayesian analysis, information on the choice of priors and Markov chain Monte Carlo settings
☒	☐	For hierarchical and complex designs, identification of the appropriate level for tests and full reporting of outcomes
☐	☒	Estimates of effect sizes (e.g. Cohen's *d*, Pearson's *r*), indicating how they were calculated

Our web collection on statistics for biologists contains articles on many of the points above.

Software and code

Policy information about availability of computer code

Data collection	TopSpin software (version 4.1.1, Bruker, USA)
Data analysis	Chenomx 9.02<br>KneadData<br>MetaPhlan 4.0.4.<br>Vegan 2.6-2<br>HUMAnN 3.6.0<br>MaAsLin 2

For manuscripts utilizing custom algorithms or software that are central to the research but not yet described in published literature, software must be made available to editors and reviewers. We strongly encourage code deposition in a community repository (e.g. GitHub). See the Nature Portfolio guidelines for submitting code & software for further information.

Data

Policy information about availability of data

All manuscripts must include a data availability statement. This statement should provide the following information, where applicable:

- Accession codes, unique identifiers, or web links for publicly available datasets
- A description of any restrictions on data availability
- For clinical datasets or third party data, please ensure that the statement adheres to our policy

Raw sequencing data and associated metadata (participant details, substrate and sampling timepoint) can be accessed through NCBI SRA project number PRJNA928249. Prior to publication this data has been made available to reviewers at https://dataview.ncbi.nlm.nih.gov/object/PRJNA928249?reviewer=m43kair2jh2rmogu3uc2vbt4f0

Research involving human participants, their data, or biological material

Policy information about studies with human participants or human data. See also policy information about sex, gender (identity/presentation), and sexual orientation and race, ethnicity and racism.

Reporting on sex and gender	Sex or gender related information was not collected for the participants providing samples for this study
Reporting on race, ethnicity, or other socially relevant groupings	Social grouping information was not collected for the participants providing samples for this study
Population characteristics	Participants self-reported as healthy with GI related diseases or recent antibiotic usage
Recruitment	Participants were recruited through a database of stool sample donors held by the model colon facility manager at the Quadram Institute
Ethics oversight	Human Research Governance Committee at the Quadram Institute (IFR01/2015) and London - Westminster Research Ethics Committee (15/LO/2169), and the trial was registered on clinicaltrials.gov (NCT02653001)

Note that full information on the approval of the study protocol must also be provided in the manuscript.

Field-specific reporting

Please select the one below that is the best fit for your research. If you are not sure, read the appropriate sections before making your selection.

☒ Life sciences ☐ Behavioural & social sciences ☐ Ecological, evolutionary & environmental sciences

For a reference copy of the document with all sections, see nature.com/documents/nr-reporting-summary-flat.pdf

Life sciences study design

All studies must disclose on these points even when the disclosure is negative.

Sample size	n=6. Sample size was determined on the basis of previous studies informing power calculations to estimate the sample size required to identify significant differences in short chain fatty acid production during in vitro fermentation
Data exclusions	None
Replication	Each donor stool was used to ferment each substrate in duplicate
Randomization	All stool samples were used to ferment all samples in a fully randomised order
Blinding	Blinding was not possible as in vitro the substrates are clearly distinct. However, given the in vitro nature of the experiments this is not expected to introduce significant bias

Reporting for specific materials, systems and methods

We require information from authors about some types of materials, experimental systems and methods used in many studies. Here, indicate whether each material, system or method listed is relevant to your study. If you are not sure if a list item applies to your research, read the appropriate section before selecting a response.

Materials & experimental systems

n/a	Involved in the study
☒	☐ Antibodies
☒	☐ Eukaryotic cell lines
☒	☐ Palaeontology and archaeology
☒	☐ Animals and other organisms
☒	☐ Clinical data
☒	☐ Dual use research of concern
☒	☐ Plants

Methods

n/a	Involved in the study
☒	☐ ChIP-seq
☒	☐ Flow cytometry
☒	☐ MRI-based neuroimaging
